# A case-control study of pattern and determinants of quality of life of patients with diabetes in a developing country

**DOI:** 10.1186/s42506-020-00061-y

**Published:** 2021-01-28

**Authors:** Ofem Enang, Ogban Omoronyia, Udeme Asibong, Agam Ayuk, Kenneth Nwafor, Annette Legogie

**Affiliations:** 1 Department of Internal Medicine, University of Calabar, Calabar, Nigeria; 2 Department of Community Medicine, University of Calabar, Calabar, Nigeria; 3 Department of Family Medicine, University of Calabar, Calabar, Nigeria

**Keywords:** Diabetes mellitus, Quality of life, Calabar, Nigeria

## Abstract

**Background:**

Globally, diabetes is a leading cause of impairment of quality of life. In the sub-Saharan African region, there is a need for studies that provide more valid assessment of effect of diabetes on quality of life (QoL). This study aimed at assessing quality of life among patients with diabetes attending a tertiary health service in Nigeria.

**Methods:**

The study design was a case-control. Diabetic cases were randomly recruited from the University of Calabar Teaching Hospital, while non-diabetic controls were civil servants and retirees. The validated and pretested WHOQoL-BREF instrument was used to assess quality of life, with higher scores indicating higher quality of life.

**Results:**

Three hundred and thirty subjects were studied, with mean ages of males and females of 55.2 ± 4.8 and 51.8 ± 6.3 years, respectively. The mean total QoL score was 75.77 ± 11.2, with no significant difference between males and females. Among male and female cases, the mean score of the physical health domain was significantly lower for cases compared with controls (*p* = 0.05). Male cases compared with controls had higher scores for the environment domain (*p* < 0.05). Older age and higher systemic blood pressure were associated with lower QoL scores for both sexes (*p* < 0.05). Unmarried status, obesity, and poor glycemic control (HbA1c > 7%) were associated with lower QoL scores (*p* < 0.05). Fasting blood sugar (FBS) level and lipid profile were not significantly correlated with QoL score in both sexes (*p* > 0.05).

**Conclusion:**

Diabetes contributes to low quality of life among males and females, with significant differences in the affected domains. Diabetes care providers should identify affected domains during clinic consultation, in order to improve provision of more effective care.

## Background

National and global health systems are ultimately aimed at preventing disease and promoting health, especially via goal-oriented improvement in longevity and quality of life [1, 2]. Chronic non-communicable diseases (NCDs) constitute a potential rate-limiting step towards attainment of the current sustainable development goals (SDGs), since their presence and degree of control is one of the greatest determinants of health-related quality of life [3].

Health-related quality of life (HRQoL), which refers to sociocultural and time-specific sense of well-being, is assessed in different areas of life, mainly comprising physical, psychological, social, and environment domains that are related to health [4]. The onset, progression, and prognosis of most chronic diseases are key determinants of HRQoL. Diabetes mellitus is one of such NCDs, which has continued to make significant contribution to high burden of disease and adverse impact on HRQoL in both developed and developing countries [5, 6]. An estimated 451 million people are diabetic, and 693 million are projected to have the chronic disease by 2045 [7]. This suggests that in the coming years and decades, HRQoL is expected to consistently decrease due to rising burden of diabetes and other NCDs.

Decreased HRQoL affects virtually all facets of human existence, including national and domestic productivity [4]. Developing countries which need to do much more productively to grow their economies may therefore be worse hit by impaired HRQoL effects of chronic diseases [6]. Best practice of diabetes care aims at attaining near-normal control of blood sugar without significantly affecting HRQoL of patients [8]. Studies in developing countries have shown high prevalence of poor glycemic control among patients with diabetes, potentially contributing to impairment in their HRQoL [9]. Also, a multi-center study among diabetic patients in Nigeria found 67.6% prevalence of poor glycemic control [10]. Still, attainment of good glycemic control demands life-long self-monitoring and care and awareness of risk of occurrence and progression of complications [9]. This potential impairment in quality of life may in turn lead to poor compliance with treatment, disease progression, and eventual poor prognostic outcome [6, 11].

Yet, in sub-Saharan Africa (SSA) where the disease is on the rapid rise with poor healthcare access, most studies of HRQoL among type 2 diabetics are descriptive cross-sectional studies, without at least comparison or control group(s), which provide more credible evidence of disease impact on QoL [12–16]. HRQoL may be affected by several other factors including poverty, insecurity, unemployment, and chronic diseases, which are also prevalent in the region. Therefore, there is a need for better understanding of level and determinants of HRQoL among patients with diabetes in the region. This will enable better improvement in quality of diabetic care services provided in developing countries [6]. The purpose of this study was to assess the pattern and determinants of HRQoL among patients with diabetes in Nigeria using a control group of healthy non-diabetics.

## Methods

### Study design and participants

This case-control study explored HRQoL among adults with type 2 diabetes in Calabar, Southern Nigeria. Primary data for cases was obtained from diabetic patients seen in medical wards and outpatient clinics in the University of Calabar Teaching Hospital (UCTH), while age/sex-matched controls were non-diabetic and normotensive civil servants and retirees. The teaching hospital is a referral center for diabetes care in the region and runs a weekly diabetes clinic. Five (5) civil service ministries were randomly selected from the existing twelve (12) ministries in Calabar to recruit the controls.

### Sampling

Sample size *n* was calculated using the formula: *n* = {*Z*_1 − α/2_√(2*P*(1 − *P*) + *Z*_1 − β_ √[P* (1 − P*) + *P* (1 − *P*)]}^2^/*D*^2^ [17] where Z_1 − α/2_ is the critical value at 5% level of significance = 1.96, *Z*_1 − β_ is the standard normal deviate corresponding to 80% power = 0.84, *P* is the proportion of general population with poor quality of life (0.184), reported in previous study in Nigeria [18], P* is the estimated proportion of diabetics with poor quality of life assuming odds ratio of 2 as important difference between the two groups = 0.33, and *D* is the effect size (P* − *P*). With assumption of 5% non-response rate, a sample size of 165 was obtained for each of the two arms, yielding a total sample size *N* of three hundred and thirty (*n* = 330) subjects. Systematic random sampling was used to recruit cases and controls, using the registers of the medical outpatient clinic and departmental civil service as the sampling frames, respectively.

### Data collection

The English version of WHOQoL-BREF instrument [19] was used in this study. It is a 26-item questionnaire comprising physical health, psychological health, social relationships, and environment domains. Two items assess overall quality of life and general health, while 7, 6, 3, and 8 items assess physical health, psychological health, social relationship, and environmental health, respectively. Each item has a 5-point Likert scale, and the mean score of items in each domain contributes to the domain score which is multiplied by four (4) to correspond to a 0 to 100 scale. Negatively phrased questions comprising items 3, 4, and 26 were reversely scored (5 = 1, 4 = 2, 3 = 3, 2 = 4, 1 = 5). Greater score indicates better quality of life and vice-versa [20]. The instrument has been validated in diverse global settings including Nigeria [21] and was pretested in this study, with yield of Cronbach’s alpha of 0.79 before use for data collection. Trained research assistants administered the questionnaire in English. Fasting blood sugar, HbA1c, triglyceride, cholesterol, HDL-c, and LDL-c were assessed using venous blood samples drawn following overnight fast by subjects. Auto analyzer (Hitachi 902) was used by trained laboratory scientist to measure these parameters, in tune with the manufacturer’s guidelines. Height (to the nearest 0.5 cm) and weight (to the nearest 0.1 kg) were assessed using standard calibrated stadiometer (SECA, Hamburg, Germany), with subjects having light clothing and not wearing any shoes. BMI (kg/m^2^) was calculated as ratio of weight (in kg) and square of height (in meters). After at least 5 min of rest, systemic blood pressure was measured by a trained nurse using desk-type analog mercury sphygmomanometer (Accoson, Dekamet, UK), with subject sitting upright and legs uncrossed. Measurement was repeated after 30 min, and the mean value was reported for both systolic and diastolic blood pressures.

### Statistical analysis

Data entry and analysis was done using SPSS version 21.0. Subject characteristics were described using measures of central tendency, dispersion, and frequency. State of glycemic control among cases was determined using HbA1c level, with subjects having 7.0% or higher were considered to have poor control and vice-versa [22]. Frequency cross-table was used to present comparison of sociodemographic, anthropometric, and clinical characteristics between cases and controls, with Chi-square or Fisher’s exact as inferential test for differences between proportions. Independent *t* test was used to compare mean values of each of the items and domains of QoL between cases and controls. Comparison of mean values of each of the domains of QoL was done for sociodemographic and clinical characteristics using independent *t* test and analysis of variance (ANOVA) for characteristics with two or more categories, respectively. Pearson correlation analysis was done to assess the relationship between continuous biochemical variables and QoL scores. The *p* value was set at 0.05.

## Results

Complete data from three hundred and thirty subjects comprising eighty-seven (87) male and seventy-eight (78) female diabetic cases and equal number of age/sex-matched controls yielded a response rate of 95.4%. The mean ages of males and females were 55.2 ± 4.8 and 51.8 ± 6.3 years, respectively. Most subjects were within the range of 51–70 years (66.0%), were married (80.9%), had at least secondary level of education (88.8%), and never smoked (72.4%) (Table 1). The commonest religion was Roman Catholic (41.2%). Being widowed, having lower level of education, previous smoking history, and consuming alcohol occasionally were significantly more common among cases compared with controls (*p* < 0.01, Table 2). The mean BMI was 26.7 ± 4.7, and most subjects (61.2%) were either overweight or obese, with significantly higher proportion among cases compared with controls (*p* < 0.01). Hypertension was found in 85.5% of cases, with mean duration of hypertension being 8.5 ± 3.6 years, ranging from 1 to 15 years. Also, among cases, the mean HbA1c was 8.46 ± 2.2% (4.40–13.60), and 29.1% had good control. There was no significant difference in level of control comparing males and females (8.61 vs. 8.30%, *p* = 0.37). Comparing cases with good and poor control of diabetes, there was no significant difference in mean scores of total (76.8 vs. 73.4), psychological (19.6 vs. 19.0), social relationship (9.46 vs. 9.38), and environment (24.6 vs. 23.6) domains of quality of life (*p* > 0.05). However, physical quality of life was significantly lower among cases with poor compared with good control of their diabetes (21.4 vs. 23.2, *p* = 0.03).

**Table 1:** Characteristics of patients with diabetes and controls in Calabar, Nigeria (*N* = 330)

Characteristic	Case (*n* = 165)	Control (*n* = 165)	Total (*n* = 330)	Chi-square
	***n*** (%)	***n*** (%)	***n*** (%)	(***p*** value)
**Sex**				
Male	87 (52.7)	87 (52.7)	174 (52.7)	1.00
Female	78 (47.3)	78 (47.3)	156 (47.3)	
**Age groups** (in years)				
≤ 40	14 (8.5)	14 (8.5)	28 (8.5)	1.00
41–50	33 (20.0)	33 (20.0)	66 (20.0)	
51–60	72 (43.6)	72 (43.6)	144 (43.6)	
61–70	37 (22.4)	37 (22.4)	74 (22.4)	
> 70	9 (5.5)	9 (5.5)	18 (5.5)	
**Marital status**				
Married	122 (73.9)	145 (87.9)	267 (80.9)	0.00
Single	7 (4.2)	7 (4.2)	14 (4.2)	
Divorced/separated	15 (9.1)	10 (6.1)	25 (7.6)	
Widowed	21 (12.7)	3 (1.8)	24 (7.3)	
**Educational level**				
Primary or less	33 (20.0)	4 (2.4)	37 (11.2)	0.00
At least secondary	132 (80.0)	161 (97.6)	293 (88.8)	
**Religion**				
Pentecostal	74 (44.8)	51 (30.9)	125 (75.8)	0.00
Orthodox	22 (13.3)	28 (17.0)	50 (15.2)	
Catholic	53 (32.1)	83 (50.3)	136 (41.2)	
Others	16 (9.8)	3 (1.8)	19 (5.8)	
**Smoking status**				
Yes (currently)	6 (3.6)	0 (0.0)	6 (1.8)	0.00
Smoked previously but stopped	57 (34.5)	28 (17.0)	85 (25.8)	
Never	102 (61.9)	137 (83.0)	239 (72.4)	
**Consume alcohol**				
Frequently	12 (7.3)	7 (4.2)	19 (5.8)	0.00
Occasionally	21 (12.7)	47 (28.5)	68 (20.6)	
Rarely	67 (40.6)	60 (36.4)	127 (38.5)	
Never	65 (39.4)	51 (30.9)	116 (35.1)	
**BMI category**				
Normal	41 (24.9)	87 (52.7)	128 (38.8)	0.00
Overweight	85 (51.5)	75 (45.5)	160 (48.5)	
Obese	39 (23.6)	3 (1.8)	42 (12.7)	
**Comorbid hypertension**				
Yes	141 (85.5)	0 (0.0)	141 (42.7)	0.00
No	24 (14.5)	165 (100)	189 (59.3)

**Table 2:** Comparison of QoL scores among female patients with diabetes and controls in Calabar (*N* = 156)

Variable	Case (*n* = 78)	Control (*n* = 78)	***p*** value
	Mean ± SD	Mean ± SD	
**General (non-domain) questions**			
1 How would you rate your quality of life?	3.64 ± 0.87	3.94 ± 0.67	0.02*
2 How satisfied are you with your health?	3.04 ± 1.04	3.72 ± 0.68	0.00*
**Physical health domain**			
3 How much physical pain prevents you from doing things?	3.47 ± 1.24	3.68 ± 0.80	0.22
4 How much do you need medical treatment to function daily?	2.59 ± 0.92	3.85 ± 1.03	0.00*
10 Do you have enough energy for everyday life?	3.22 ± 0.95	3.62 ± 0.87	0.01*
15 How well are you able to go around?	3.35 ± 0.74	3.13 ± 1.06	0.14
16 How satisfied are you with your sleep?	2.95 ± 1.01	3.82 ± 0.86	0.00*
17 How satisfied are you with ability to perform daily activities?	3.36 ± 1.02	3.42 ± 0.59	0.63
18 How satisfied are you with your capacity for work?	3.29 ± 0.87	3.37 ± 0.74	0.55
Subtotal score for physical health	22.23 ± 5.04	24.88 ± 3.36	0.00*
**Psychological health domain**			
5 How much do you enjoy life?	2.49 ± 1.14	2.40 ± 0.81	0.57
6 To what extent do you feel your life to be meaningful?	2.35 ± 0.82	2.44 ± 0.59	0.44
7 How well are you able to concentrate?	2.82 ± 1.02	3.26 ± 0.87	0.01*
11 Are you able to accept your bodily appearance?	3.62 ± 1.08	4.12 ± 0.82	0.00*
19 How satisfied are you with yourself?	3.69 ± 0.74	3.96 ± 0.89	0.04*
26 How often do you have negative feelings such as mood, despair, anxiety, and depression?	4.31 ± 0.71	3.21 ± 0.92	0.00*
Subtotal score for psychological health	19.27 ± 1.46	19.37 ± 1.76	0.69
**Social Relationship Domain**			
20 How satisfied are you with your personal relationships?	3.47 ± 1.11	3.79 ± 0.95	0.05
21 How satisfied are you with the support you get from friends?	2.78 ± 1.27	2.49 ± 1.13	0.13
22 How satisfied are you with your sex life?	3.21 ± 1.11	3.64 ± 1.00	0.01*
Subtotal score for social relationship	9.46 ± 2.62	9.92 ± 2.29	0.24
**Environment domain**			
8 How safe do you feel in your daily life?	3.22 ± 0.75	3.26 ± 0.55	0.72
9 How healthy is your physical environment?	3.28 ± 0.70	4.09 ± 0.93	0.00*
12 Have you enough money to meet your needs?	2.68 ± 0.71	3.05 ± 0.82	0.00*
13 How available to you is the information that you need daily?	2.72 ± 0.88	2.38 ± 0.89	0.02*
14 To what extent do you have the opportunity for leisure?	2.91 ± 0.74	2.00 ± 1.02	0.00*
23 How satisfied are you with condition of your living space?	2.86 ± 0.99	2.87 ± 1.36	0.95
24 How satisfied are you with your access to health?	3.14 ± 0.96	3.04 ± 1.05	0.53
25 How satisfied are you with your transport?	3.00 ± 0.91	2.96 ± 0.97	0.04*
Subtotal score for environment	23.81 ± 4.13	23.65 ± 4.50	0.82
**Domain-based total QoL score**	74.77 ± 11.4	77.83 ± 10.1	0.08

*Significant *p* value

The mean QoL score for all (male and female) patients with diabetes was 75.77 ± 11.2, with no significant difference between males and females (77.1 ± 10.6 vs. 76.3 ± 10.4, *p* > 0.05). Among females, the mean score of the physical health domain was significantly lower for cases compared with controls (*p* < 0.01, Table 2). The mean scores for psychological health, social relationship, and environment domains were not significantly different between cases and controls (*p* > 0.05). However, the mean scores for one of the three items in social relationship and four of the eight items in environment domains were significantly lower among cases compared with controls (*p* < 0.05).

Table 3 compared the mean scores of each item and domain of QoL among male cases and controls. Physical health and social relationship domains had significantly lower mean sum scores for cases compared with controls (*p* < 0.05). Four of seven, one of six, one of three, and two of eight items were significantly lower among cases compared with controls for physical, psychological health, social relationship, and environment domains, respectively (*p* < 0.05). Psychological health and environment domains had mean sum scores that were higher among cases compared with controls, but statistical significance was found only for the environment domain (*p* < 0.05).

**Table 3:** Comparison of QoL scores among male patients with diabetes and controls in Calabar (*N* = 174)

Variable	Case (*n* = 87)	Control (*n* = 87)	***p*** value
	Mean ± SD	Mean ± SD	
**General (non-domain) questions**			
1 How would you rate your quality of life?	3.79 ± 0.89	3.55 ± 0.57	0.03*
2 How satisfied are you with your health?	3.38 ± 0.97	3.52 ± 0.86	0.32
**Physical health domain**			
3 How much physical pain prevents you from doing things?	4.17 ± 0.99	3.72 ± 0.87	0.00*
4 How much do you need medical treatment to function daily?	3.03 ± 1.01	4.24 ± 0.78	0.00*
10 Do you have enough energy for everyday life?	3.14 ± 0.82	3.38 ± 0.85	0.06
15 How well are you able to go around?	3.59 ± 0.62	3.10 ± 0.76	0.00*
16 How satisfied are you with your sleep?	2.97 ± 1.07	3.45 ± 0.82	0.00*
17 How satisfied are you with ability to perform daily activities?	3.00 ± 1.09	3.17 ± 0.70	0.22
18 How satisfied are you with your capacity for work?	3.17 ± 0.96	3.52 ± 0.78	0.01*
Subtotal score for physical health	23.07 ± 4.70	24.59 ± 3.57	0.02*
**Psychological health domain**			
5 How much do you enjoy life?	2.45 ± 0.62	2.55 ± 0.68	0.30
6 To what extent do you feel your life to be meaningful?	2.45 ± 0.77	2.69 ± 0.70	0.03*
7 How well are you able to concentrate?	3.17 ± 0.88	3.14 ± 0.63	0.77
11 Are you able to accept your bodily appearance?	3.34 ± 1.03	3.72 ± 0.87	0.10
19 How satisfied are you with yourself?	3.62 ± 0.77	3.55 ± 0.97	0.60
26 How often do you have negative feelings such as mood, despair, anxiety, and depression?	4.48 ± 0.73	3.76 ± 0.82	0.00*
Subtotal score for psychological health	19.52 ± 2.17	19.41 ± 1.95	0.74
**Social relationship domain**			
20 How satisfied are you with your personal relationships?	3.24 ± 1.01	3.76 ± 0.78	0.00*
21 How satisfied are you with the support you get from friends?	2.93 ± 1.35	3.14 ± 1.05	0.26
22 How satisfied are you with your sex life?	3.24 ± 1.23	3.30 ± 1.42	0.78
Subtotal score for social relationship	9.41 ± 2.74	10.20 ± 2.32	0.04*
**Environment domain**			
8 How safe do you feel in your daily life?	3.24 ± 0.73	3.41 ± 0.62	0.10
9 How healthy is your physical environment?	3.28 ± 0.74	3.76 ± 0.90	0.00*
12 Have you enough money to meet your needs?	3.00 ± 0.84	2.69 ± 0.70	0.01*
13 How available to you is the information that you need daily?	2.93 ± 0.79	2.14 ± 0.94	0.00*
14 To what extent do you have the opportunity for leisure?	2.72 ± 0.87	2.48 ± 1.17	0.13
23 How satisfied are you with condition of your living space?	3.10 ± 0.81	3.62 ± 0.67	0.00*
24 How satisfied are you with your access to health?	3.17 ± 1.09	2.07 ± 1.24	0.00*
25 How satisfied are you with your transport?	3.31 ± 0.84	3.07 ± 0.64	0.04*
Subtotal score for environment	24.76 ± 3.97	23.24 ± 3.58	0.01*
**Domain-based Total QoL score**	76.76 ± 11.7	77.44 ± 9.53	0.68

*Significant *p* value

Table 4 assesses the relationship between sociodemographic characteristics and QoL among cases. Among females, the mean score of the physical health domain was significantly lower among those older than 50 years (*p* < 0.05). Also, the mean score of social relationship was significantly lower for cases who were unmarried and those who never consumed alcohol (*p* < 0.05). The level of education and religion were not significantly associated with the mean scores of all the domains (*p* > 0.05). Among males, older cases had lower QoL scores for all domains, but statistical significance was found only for the environment domain (*p* < 0.05). Marital status, level of education, religion, and satisfaction about sexual life were not significantly associated with the mean QoL scores for all the domains (*p* > 0.05, Table 3).

**Table 4:** Relationship between characteristics and QoL among cases in Calabar (*N* = 165)

Sex	Variable	Total mean ± SD	Phys. Health Mean ± SD	Psych. Health mean ± SD	Soc. Rel. mean ± SD	Environment mean ± SD
Females (*n* = 78)	**Age group** (in years)					
	≤ 50	78.86 ± 12.26	24.33 ± 5.34	19.17 ± 1.44	9.76 ± 2.77	25.10 ± 3.90
	> 50	72.35 ± 10.17	20.69 ± 4.20	19.33 ± 1.48	9.29 ± 2.53	23.04 ± 4.11
	*t* test *p* value	0.01*	0.00*	0.66	0.44	0.03*
	**Marital status**					
	Married	76.85 ± 11.67	22.72 ± 5.29	19.47 ± 1.64	10.19 ± 2.67	24.47 ± 3.91
	Unmarried	70.36 ± 9.46	21.20 ± 4.39	18.84 ± 0.85	7.92 ± 1.71	22.40 ± 4.31
	*p* value	0.02*	0.22	0.07	0.00*	0.04*
	**Educational level**					
	Primary or none	73.76 ± 9.46	21.33 ± 4.58	19.52 ± 1.47	9.38 ± 1.77	23.52 ± 2.91
	At least secondary	75.14 ± 12.05	22.56 ± 5.20	19.18 ± 1.45	9.49 ± 2.88	23.91 ± 4.52
	*p* value	0.64	0.34	0.35	0.87	0.72
	**Religion**					
	Pentecostal	76.69 ± 13.99	23.88 ± 5.68	19.81 ± 1.30	9.77 ± 3.02	23.23 ± 4.95
	Orthodox	71.60 ± 7.37	19.30 ± 2.87	19.20 ± 1.03	8.30 ± 2.36	24.80 ± 4.37
	Catholic	71.97 ± 9.69	20.71 ± 4.23	18.51 ± 1.25	9.54 ± 2.61	23.20 ± 3.42
	Others	73.42 ± 11.07	21.30 ± 3.07	19.17 ± 1.07	9.57 ± 0.54	23.74 ± 3.54
	*p* value	0.12	0.09	0.14	0.51	0.67
**Males** (*n* = 87)	**Age group** (in years)					
	≤50	82.50 ± 10.80	24.83 ± 4.88	20.33 ± 2.28	10.17 ± 2.01	27.17 ± 3.00
	> 50	75.26 ± 11.50	22.61 ± 4.63	19.30 ± 2.11	9.22 ± 2.88	24.13 ± 3.97
	*t* test (*p* value)	0.02*	0.08	0.07	0.19	0.00*
	**Marital status**					
	Married	76.70 ± 11.24	23.35 ± 4.29	19.39 ± 2.26	9.22 ± 2.79	24.74 ± 4.05
	Unmarried	77.00 ± 13.60	22.00 ± 6.20	20.00 ± 1.78	10.17 ± 2.48	24.83 ± 3.73
	*t* test (*p* value)	0.92	0.29	0.29	0.19	0.93
	**Educational level**					
	Primary or none	75.25 ± 8.34	21.50 ± 5.25	19.75 ± 0.45	10.25 ± 3.57	23.75 ± 2.38
	At least secondary	77.00 ± 12.15	23.32 ± 4.64	19.48 ± 2.33	9.28 ± 2.59	24.92 ± 4.16
	*t* test (*p* value)	0.63	0.22	0.69	0.26	0.35
	**Religion**					
	Pentecostal	75.38 ± 12.56	22.81 ± 4.86	19.06 ± 2.61	8.94 ± 2.69	24.56 ± 4.30
	Orthodox	71.75 ± 8.94	20.00 ± 3.91	18.75 ± 1.14	10.75 ± 3.17	22.25 ± 2.38
	Catholic	78.67 ± 9.87	23.83 ± 4.50	20.33 ± 0.49	8.50 ± 2.20	26.00 ± 3.61
	Others	73.49 ± 10.07	21.62 ± 2.29	19.10 ± 1.07	9.17 ± 1.61	23.44 ± 2.91
	*p* value	0.09	0.19	0.11	0.21	0.08

*Significant *p* value

Table 5 shows the relationship between clinical, laboratory parameters, and QoL scores among male and female cases. In both sexes, QoL score was significantly lower among those who had abnormal BMI (overweight or obese) and poor glycemic control (HbA1C ≥ 7.0%) (*p* < 0.05). Lower QoL was also found among hypertensive compared with normotensive subjects, though this difference was not statistically significant (*p* > 0.05). However, systemic blood pressure was significantly and indirectly correlated with QoL for both sexes. Duration of diabetes and hypertension were indirectly correlated with QoL score but statistical significance was found only for males. Fasting blood sugar (FBS) level and lipid profile were not significantly correlated with QoL score in both sexes.

**Table 5:** Relationship between clinical parameters and QoL score among cases in Calabar, Nigeria (*N* = 165)

Variable	Female QoL score mean + SD	Male QoL score mean + SD
	(*n* = 78)	(*n* = 87)
**Comorbid hypertension**		
Yes	73.9 ± 11.2	76.00 ± 13.6
No	78.6 ± 11.6	76.85 ± 11.5
*p* value	0.15	0.84
**BMI**		
Normal	77.3 ± 10.2	76.5 ± 11.1
Overweight or obese	71.6 ± 10.8	70.1 ± 9.62
*p* value	0.03*	0.02*
**HbA1c status**		
Good control (< 7.0%)	75.1 ± 9.3	76.6 ± 9.8
Poor control (≥ 7.0%)	66.7 ± 9.6	68.2 ± 9.1
*p* value	0.00*	0.00*
**Duration of diabetes**		
Correlation coef. (*p* value)	− 0.06 (0.58)	− 0.32 (0.00) *
**Duration of hypertension**		
Correlation coef. (*p* value)	− 0.06 (0.64)	− 0.29 (0.01) *
**Systolic blood pressure**		
Correlation coef. (*p* value)	− 0.22 (0.05)	− 0.21 (0.05)
**Diastolic blood pressure**		
Correlation coef. (*p* value)	− 0.24 (0.03) *	− 0.24 (0.03) *
**Mean blood pressure**		
Correlation coef. (*p* value)	− 0.24 (0.03) *	− 0.18 (0.09)
**Fasting blood sugar**		
Correlation coef. (*p* value)	0.17 (0.14)	0.19 (0.11)
**Total cholesterol**		
Correlation coef. (*p* value)	-0.02 (0.85)	− 0.07 (0.73)
**HDL cholesterol**		
Correlation coef. (*p* value)	0.13 (0.25)	− 0.29 (0.79)
**LDL cholesterol**		
Correlation coef. (*p* value)	0.17 (0.14)	− 0.19 (0.11)
**Triglycerides**		
Correlation coef. (*p* value)	− 0.24 (0.03) *	− 0.21 (0.05)

*Significant *p* value

## Discussion

This study aimed at assessing health-related quality of life (HRQoL) among men and women with diabetes and comparing it with their controls in a tertiary health service in Nigeria. There was no significant difference in the overall quality of life between cases and controls in both sexes (*p* > 0.05). Diabetic women compared with controls had lower HRQoL score for physical health domain only, while diabetic men compared with controls had lower HRQoL for both physical health and social relationship domains. This implies that diabetes has significant impact on physical health for both sexes and additional impact on the social relationship among men in the study setting. Similar studies in Canada [23], Saudi Arabia [24], Israel [25], Spain [26], and Bangladesh [27] found significantly lower physical and mental health QoL scores among cases compared with controls (*p* < 0.05). These findings are however not in line with a similar study in India [28], where there was no significant difference in overall HRQoL score and scores for each of the domains comparing diabetic and non-diabetic subjects. Differences in findings may be due to differences in ethnicity and sociocultural determinants of health and well-being in different settings, as well as potential difference in perception of health status and response to the different instruments used for assessment of HRQoL [26, 29]. Physical health effects may result from complications which progressively debilitate and prevent effective performance of daily activities. Also, potential presence of adverse sexual dysfunction, as well as adherence to nutritional and other lifestyle restrictions required for better diabetes care, may be the reasons behind the higher impact on the social relationship in men with diabetes than women in the study setting [11].

In this study, the QoL score for the environment domain was higher among cases compared with controls, but with statistical significance only among men. This finding may be because cases due to their disease condition may be having better access to locally available healthcare services, health information, and awareness of the need and means of relaxation and leisure for better well-being, compared with controls [30, 31]. This may also be a reflection of poor knowledge and non-practice of healthy living, including poor attitude to health seeking at least for regular medical check-up among apparently healthy individuals in the general population [32].

Increasing age was associated with lower QoL among cases, with statistical significance for environment domain for both sexes and physical domain for females only. With increasing age, the functional capacity may be significantly impaired, especially among individuals who had previously lived sedentary lifestyle [33, 34]. This impairment may contribute to low HRQoL especially among older people with chronic diseases. Unlike the more developed settings, older people in the less developed study setting do not have improved access to healthcare, housing, security, and recreation, and therefore have lower QoL score in the environment domain. This may be a reflection of poor means of transportation, worsening insecurity, poor retirement benefit, and lack of health insurance and requisite infrastructure for healthful living of older people in developing countries [35]. This suggest that general economic and infrastructural development through good and consistent political will of government play key roles in determining the overall quality of life of patients with diabetes in developing countries.

Unlike males, unmarried females with diabetes had significantly lower QoL than married female cases. A similar study in Greece [36] found unmarried status to be a significant predictor of lower HRQoL among patients with diabetes of both sexes. This suggests that in the study setting, marital status may be key determinant of QoL among diabetic women, perhaps via the socioculturally driven potential stigma and perception of unfulfilled life among older females who are unmarried [37]. Counseling during clinic sessions including active involvement in family and suitable social support groups and consistent media-based societal reorientation may be useful for cushioning this effect of unmarried status on HRQoL among women with diabetes in developing countries [35].

The presence of comorbid hypertension was not significantly associated with HRQoL for both sexes in this study. This is in contrast with reports from studies in Greece [36], and Spain [38], where presence of hypertension and other comorbidities was associated with lower HRQoL scores. However, in this study, subjects’ systemic blood pressures were found to be indirectly correlated with HRQoL. This finding suggests that blood pressure control rather than presence of hypertension was key to maintaining or improving HRQoL among patients with diabetes in the study setting. Uncontrolled blood pressure increases cost of care, as well as risk of anxiety, depression, and multiple end-organ complications which ultimately impair QoL [39, 40]. Also, laboratory measurements including FBS and HbA1c were not associated with HRQoL in this study. This is not in agreement with the result of a similar study in Saudi Arabia [24], where patients with uncontrolled/poorly controlled blood sugar levels were found to have significantly lower HRQoL scores compared with controlled patients. Patients with diabetes in this study setting may be less aware or be nonchalant about the potential health implications of poor blood sugar control. Despite awareness of risk of complications, as long as there are no current symptoms of the disease or medication side-effects, patients may continue with unhealthy habits such as excessive alcohol consumption, perhaps due to false perception of good QoL [41]. More superior prospective study designs will be required for better understanding of the factors and relationship between glycemic control and HRQoL in the study setting.

This study is one of the few researches utilizing a control study group to assess impact of diabetes on QoL in sub-Saharan Africa, where the chronic disease is rapidly rising. The findings provide useful data for endocrinologist, clinical psychologist, social workers, and other members of multidisciplinary teams involved in optimum diabetes care. The study also reveals common and gender-based differences in the effect of sociodemographic, clinical, and laboratory factors on QoL among patients with diabetes in a developing country setting. For instance, older age and poorly controlled systemic blood pressure are factors that commonly impaired the QoL for both sexes. However, while the effect of marital status mainly applied to females, the duration of comorbid hypertension mainly applied to males. These findings may be useful for improvement of the quality of counseling, as well as updating existing policies and guidelines for improvement in best practice of diabetes care in developing countries [42].

### Study limitations

A key limitation of this study was the assessment of hypertension as the only common comorbid disease among diabetic cases. Presence of comorbidities among diabetic cases may have contributed to overestimation of impairment in HRQoL attributable to diabetes. Therefore, assessment of comorbid presence of other chronic diseases (such as cancer, COPD, arthritis, peptic ulcer, and HIV) may have been useful for better understanding of the dynamics of QoL among patients with diabetes in the study setting [43]. Also, inference of causality between identified factors of QoL among patients with diabetes will require more extensive studies with superior study design. This may include experimental design which will assess QoL effects of intervening on identified factors, as well as longitudinal studies in view of potential changes of HRQoL with time in the same individuals [44].

## Conclusion

There is suboptimal QoL among male and female patients with diabetes. There was no significant difference in the overall QoL between diabetics and controls. However, the physical domain (for both sexes) and social domain (for males only) were significantly impaired among patients with diabetes compared with the controls. Poor control of blood pressure rather than comorbid presence of hypertension further worsens QoL among diabetics. Also, there were common as well as gender-based differences in factors associated with QoL among patients with diabetes, with implications for improvement in best diabetes care in developing countries. Healthcare workers’ adherence to best practice guidelines for diabetes care and improvement in healthcare access may significantly improve physical health and social relationship QoL of patients with diabetes in the study setting. Further research on QoL among diabetics should be conducted with inclusion of key chronic diseases and social support, which potentially affects QoL. Qualitative research is also recommended for better understanding of the role of psychosocial factors and coping strategies on the QoL among patients with diabetes.

## Data Availability

The corresponding author will make available data in this study upon request.

## Abbreviations

ANOVA: Analysis of variance
BMI: Body mass index
COPD: Chronic obstructive pulmonary disease
FBS: Fasting blood sugar
HDL-c: High density lipoprotein cholesterol
HIV: Human immunodeficiency virus
HRQoL: Health-related quality of life
LDL-c: Low density lipoprotein cholesterol
NCD: Non-communicable disease
QoL: Quality of life
SDGs: Sustainable development goals
UCTH: University of Calabar Teaching Hospital
WHOQoL: World Health Organization Quality of Life
